# Development of Designer Transcription Activator-Like Effector-Based Plant Growth Regulator for Higher Yield in Rice

**DOI:** 10.3389/fpls.2022.924645

**Published:** 2022-06-14

**Authors:** Yongchao Tang, Chunlian Wang, Fujun Wang, Man Li, Yanli Fang, Zhiyuan Ji, Kaijun Zhao

**Affiliations:** ^1^National Key Facility for Crop Gene Resources and Genetic Improvement, Institute of Crop Sciences, Chinese Academy of Agricultural Sciences, Beijing, China; ^2^Rice Research Institute, Guangdong Academy of Agricultural Sciences, Guangzhou, China

**Keywords:** designer TALE, growth regulators, rice yield, *OsNOG1*, *Xoo*, genetically engineered bacteria

## Abstract

Recent studies have shown that reprogramming of gene expression in a genome can induce the production of proteins enabling yield increase. The transcription activator-like effectors (TALEs) from several species of bacterial *Xanthomonas* have been extensively studied, and a series of research tools, such as genome editing tool TALENs and gene expression activators, have been developed based on the specific protein–nucleic acid recognition and binding mechanisms of TALEs. In this proof-of-principle study, we designed and constructed a designer TALE (dTALE), designated as dTALE-NOG1, to specifically target the promoter of *OsNOG1* gene in rice, and demonstrated that this dTALE can be used as a new type of plant growth regulator for better crop growth and harvest. In doing so, the dTALE-NOG1 was transferred into the non-pathogenic *Xanthomonas oryzae* pv. *oryzae* (*Xoo*) strain PH to generate a genetically engineered bacteria (GEB) strain called PH-dtNOG1. Functional verification showed that dTALE-NOG1 could significantly induce the expression of *OsNOG1*. By spraying cell suspension of PH-dtNOG1 on the rice plants during the tillering stage, the transcription level of *OsNOG1* was highly enhanced, the grain number of rice plants was increased by more than 11.40%, and the grain yield per plant increased by more than 11.08%, demonstrating that the dTALE-NOG1 was highly effective in enhancing rice yield. This work provided a new strategy for manipulating agronomical traits by reprogramming gene expression in a crop genome.

## Introduction

Food security has always been a major issue for human beings, which has become even more prominent during the COVID-19 pandemic (Stark, 2021). Over the past century, crop breeding and commercialization of new varieties have brought about a tremendous increase in food productivity. However, the yield of major crops seems to be approaching a ceiling in the recent decade (Mann, 1999; Zhang et al., 2014). As such, feeding the huge and ever-growing human population has become a bigger challenge due to the global climate change along with frequent emergences of pests/pathogens and other environmental hazards.

Basically, crop breeding relies on genetic recombination to introduce useful genes/alleles into elite germplasms, which is not only time-consuming but also expensive. Particularly, in recent times and future, it is getting harder and harder to generate and select a significantly improved genotype of major crops because favorable alleles for most simply inherited traits are already prevalent and often fixed in elite germplasms (Varshney et al., 2012; Ansari et al., 2017). On the other hand, transgenic technology of plants, especially food crops, is still strictly limited in the application of agricultural production. Therefore, new technologies that can genetically manipulate the agronomic performance of current crop varieties without the need to change their genome are highly desired; we call such technology crop agronomic performance manipulation epigenetically (CAPME). Recently, the RNA viral transfection (RVT)-based CAPME technology has been developed, which can fine-turn the crop agronomic traits by reprogramming the plant genome through transient viral induction that was delivered to the crop through aerial spray (Torti et al., 2021).

The grain yield of rice (*Oryza sativa* L.), a staple crop feeding nearly half of the world’s population (Birla et al., 2017), is a complex agronomic trait composed of several factors, including effective tiller number, grain number per panicle, and grain weight, and among them panicle formation is the key factor (Khush, 1995). Studies have revealed that *OsNOG1* gene encodes an enoyl-CoA hydratase/isomerase protein that enhances the activity of panicle meristem and increases the number of spikelets by decreasing the jasmonic acid (JA) content in rice (Huo et al., 2017). Consistent with this, the overexpression of *OsNOG1* significantly increases rice yield *via* the increase of grain number per panicle (Huo et al., 2017). Therefore, we speculate that induction of *OsNOG1* expression has the potential to increase rice yield.

Transcription activator-like effectors (TALEs) are a class of proteins identified in plant pathogenic *Xanthomonas* spp., including *Xanthomonas oryzae* pv. *oryzae* (*Xoo*), the causal agent of bacterial blight, a worldwide disease of rice (Boch and Bonas, 2010). The *Xanthomonas* pathogens inject their TALEs through the type-III secretion system (T3SS) into plant cells primarily to activate the expression of host genes that contribute to pathogen growth (Boch and Bonas, 2010), but they occasionally trigger the executor gene-mediated disease resistance in rice (Ji et al., 2022). TALEs are structurally conserved and function as eukaryotic transcription factors in inducing gene expression (Cornelis, 2006). A typical TALE in nature usually contains a secretion domain (SD), a chaperone binding domain (CBD), a DNA binding domain (DBD), multiple nuclear localization signals (NLS), and an acid transcription activation domain (TAD). The DBD consists of a variable number of 33–35 amino acid repeats that are nearly identical, except for the two variable amino acids at positions 12 and 13, designated as repeat variable di-residues (RVDs). Each RVD specifically recognizes one nucleotide, and the DNA target sequence of a given TALE is determined by the combination of a number and composition of the RVDs in the repeats (Boch et al., 2009; Moscou and Bogdanove, 2009). Based on the codes between RVDs and their recognized nucleotides (Boch et al., 2009), the central repeat region or DNA-binding domain of a TALE can be designed and modularly assembled, and this has allowed for broad applications in genome and epigenome engineering, including genome editing, transcriptional activation, transcriptional repression, and live visualization of chromatin dynamics (Anderson et al., 2020).

Here, we report the development of another CAPME technology based on the designer TALE (dTALE). A dTALE targeting the rice *OsNOG1* gene, designated as dTALE-NOG1, was constructed and transferred into the non-pathogenic *Xoo* strain PH (Ji et al., 2016) to generate the genetically engineered bacteria (GEB) strain, designated as PH-dtNOG1, that can deliver the dTALE-NOG1 into rice cells. We showed that the expression of *OsNOG1* was successfully induced by PH-dtNOG1, and rice yield was increased by simply spraying rice plants with cell suspension of PH-dtNOG1. This CAPME strategy does not involve any transgene in plants, and so it is safer and more reliable. This work provides a new strategy for promoting crop growth and increasing yield by a CAPME technology, with a significance for agricultural production and global food security.

## Materials and Methods

### Plants Materials and Experimental Conditions

The rice plants of *japonica* varieties Nipponbare (Nip), Jinjing 818 (JJ818), and *indica* varieties Guichao No.2 (GC2), R007 were planted in the screenhouse of Institute of Crop Science of Chinese Academy of Agricultural Sciences, Beijing, China. The growth season is from May to October, and the rice plants were managed with regular water and fertilizer management and natural lighting. The *Nicotiana benthamiana* plants were planted in phytotron with 14 h light (20,000 Lux) and 10 h dark, 25°C, relative humidity 70%, and watered with fertilizer once a week.

### Designer Transcription Activator-Like Effector Target Selection and Gene Sequence Analysis

The dTALE targets in promoters of *OsHYR* (LOC4331412), *OsSUT2* (LOC9266530), and *OsNOG1* (LOC4324637) were selected by considering the number of repeat units (Boch and Bonas, 2010; Zheng et al., 2014), distance to the start codon (Grau et al., 2013), distance to the transcription start site (TSS; Antony et al., 2010), sequence specificity in the rice genome, and coverage with other transcription regulatory elements (Brückner et al., 2015). One target was selected for each gene.

The complete sequences of *OsNOG1* in all rice varieties used in this study were amplified using 2 × Rapid Taq Master Mix (P222, Vazyme) with primers No. 1–10 (Supplementary Table 1) and sequenced. The alignment was generated by SnapGene software (from Insightful Science^1^) using MUSCLE method with default parameter settings. Promoters of *OsHYR*, *OsSUT2*, and *OsNOG1* were analyzed by New PLACE.^2^

### Construction of Vectors

**FIGURE 1:**
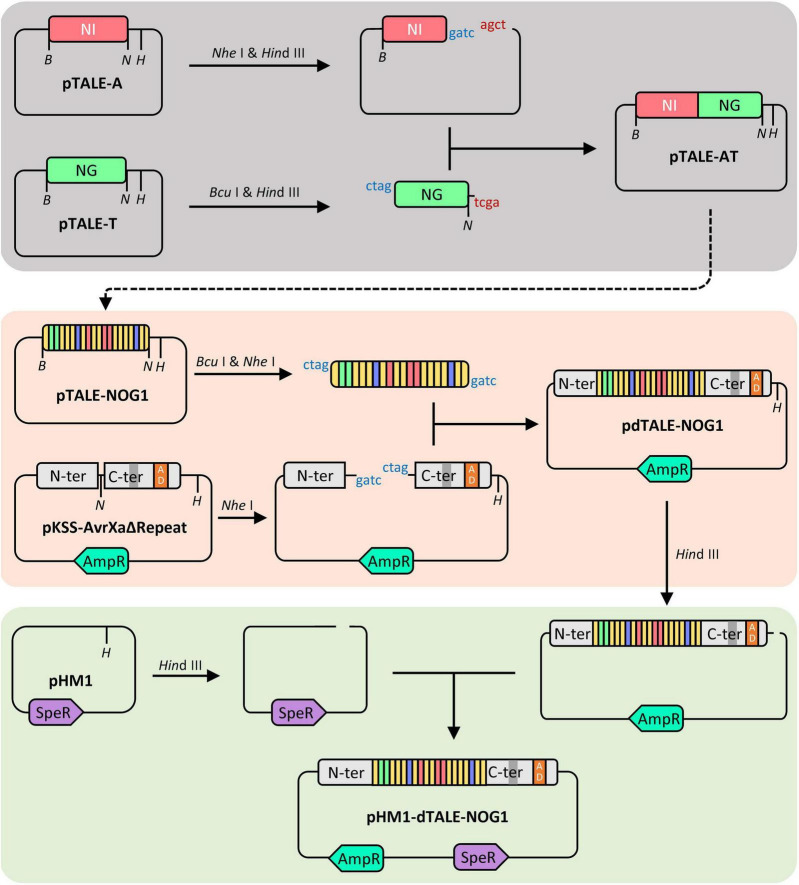
Schematic diagram of dTALE vector construction. The process of dTALE vector assembly. Based on 4 pTALE unit vectors (e.g., pTALE-A), the pTALE-NOG1 was generated by several rounds of digestion and ligation. The RVDs repeats coding region from pTALE-NOG1 was cut-off and fused into pKSS-AvrXaΔRepeat, resulting in pdTALE-NOG1. The ligation of pHM1 and pdTALE-NOG1 generated the final dTALE transformation vector pHM1-dTALE-NOG1. Restriction enzyme cutting sites of *Bcu*I, *Nhe*I, and *Hin*dIII are represented by the letter *B*, *N*, and *H*, respectively. *AmpR* codes β-lactamase for ampicillin resistance and *SpeR* codes spectinomycin adenylytransferase for spectinomycin resistance.

**FIGURE 2:**
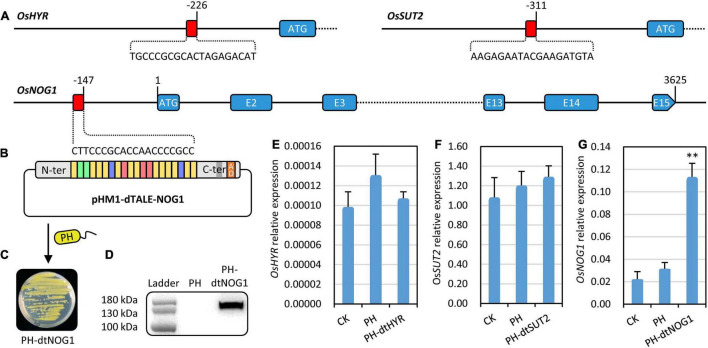
Targets of dTALEs and target gene responses to the genetically engineered bacteria (GEB) strains. **(A)** dTALE target sites in promoters of *OsHYR*, *OsSUT2*, and *OsNOG1*. The target sites were presented by red rectangle and the nucleotides were shown. The coding region or exons were shown by blue rectangles. **(B)** The schematic diagram of the construct pHM1–dTALE-NOG1, which was transferred into the non-pathogenic *Xoo* strain PH and resulted in GEB strain PH-dtNOG1. **(C)** PH-dtNOG1 grown on NA (Spe and Amp) plate. **(D)** Protein of dTALE-NOG1 expressed in PH-dtNOG1 detected by Western blot with PH as a negative control. **(E–G)** Expression of *OsHYR*, *OsSUT2*, and *OsNOG1* in rice leaves injected with PH-dtHYR, PH-dtSUT2, and PH-dtNOG1, respectively. *OsUbq* (LOC4332169) was used as the reference gene for qRT-PCR. Data values were represented as mean with error bars representing standard deviation (*n* = 3). The significance of the difference was tested by *t*-test. ^**^P < 0.01.

Three vectors for expressing dTALEs targeting promoters of the above three genes were assembled using an improved unit assembly method (Huang et al., 2011; Zheng et al., 2014). A, T, C, and G were respectively identified by RVDs NI, NG, HD, and NN. Based on the one-unit vectors (pTALE-A, pTALE-T, pTALE-C, and pTALE-G), the pTALE-NOG1, including 20 RVDs repeats coding fragments was generated by several rounds of digestion and ligation. The digestions were performed by using FastDigest *Nhe*I (FD0973, Thermo Scientific) and FastDigest *Bcu*I (FD1254, Thermo Scientific) with FastDigest *Hin*dIII (FD0504, Thermo Scientific), respectively. The ligation was achieved by using T4 DNA Ligase (2011A, Takara). The assembled DNA fragment coding the designed RVDs repeats was cut-off from pTALE-NOG1 by *Bcu*I and *Nhe*I and inserted into the *Nhe*I site of pKSS-AvrXaΔRepeat, an expression vector containing the N-segment and C-segment including DYKDDDDK tag and TAD of TALE, resulting in a vector designated as pdTALE-NOG1. The pdTALE-NOG1 was linearized and ligated into the *Hin*dIII site of the cosmid vector pHM1 (Gu et al., 2005; Figure 1). The final dTALE transformation vector pHM1–dTALE-NOG1 (Figure 1) was transferred into the *Xoo* strain PH, a PXO99^A^ none TALEs mutant, by electroporation, resulting in the engineered *Xoo* strain PH-dtNOG1 (Figure 2C). The other two dTALEs were constructed by the same process. Every step of the vector construction process was validated by Sanger sequencing. Since pHM1 contains spectinomycin adenylyltransferase and pKSS-AvrXaΔRepeat contains β-lactamase (Figure 1), the PH-dt strains, containing the corresponding dTALE, were cultured with NA medium (1.0% sucrose, 0.5% polypeptone, 0.1% yeast extract, 0.3% beef extract, pH 6.8) with ampicillin and spectinomycin added.

The activator, p1305-dTALE-NOG1, was generated by inserting the coding sequence of dTALE-NOG1 into *Nco*I and *Eco*72I sites of pCAMBIA1305. The reporter, pBI121-NOG1pro:GUS, was generated by inserting the promoter sequence of *OsNOG1* of JJ818 and GC2 (amplified using primers No. 11–12, Supplementary Table 1) into *Hin*dIII and *Sma*I sites of pBI121 using ClonExpress^®^ II One Step Cloning Kit (C112, Vazyme). These vectors were validated by Sanger sequencing.

### Designer Transcription Activator-Like Effector-NOG1 Protein Detection by Western Blot

A single colony of PH or PH-dtNOG1 was cultured in 3 mL of NA medium (spectinomycin and ampicillin added) at 200 RPM at 28°C for 24 h until turbidity. The bacteria culture was centrifuged at 12,000 RPM for 2 min to collect the bacteria and then 150 μL Solution I (25 mM pH 8.0 Tris–HCl, 10 mM EDTA, 50 mM Glucose) was added. After suspension, 30 μL lysozyme was added, mixed well, and let to stand for 5 min at room temperature. Then 200 μL of 1 × SDS loading buffer, which is precooled at 4°C, was added to the bacteria suspension, mixed well, and placed on ice. Then the suspension was for boiling water bath for 10 min, ice bath for 5 min, and centrifuged at 12,000 RPM for 1 min. Then 15 μL supernatant was used for SDS-PAGE electrophoresis and Western blot with ProteinFind^®^ Anti-DYKDDDDK Mouse Monoclonal Antibody (HT201, TransGen), ProteinFind^®^ Goat Anti-Mouse IgG (H + L), HRP Conjugate (HS201, TransGen), and Immobilon™ Western Chemiluminescent HRP Substrate (WBKL S0500, MILLIPORE) according to the product instructions. The above process was repeated independently at least three times.

### Transient Transformation and GUS Staining of *N*icotiana *benthamiana*

The *Agrobacterium tumefaciens* strain GV3101, containing the corresponding plasmid, was cultured on LB medium (rifampicin and kanamycin added). Single clones were selected and transferred into 2 mL medium for culturing at 200 RPM at 28°C overnight. Then 100 μL bacterial suspension culture was transferred into 5 mL of LB medium (rifampicin and kanamycin added) with 2 μL 0.1 M acetosyringone (AS) and 100 μL 0.5 M 2-(N-morpholino) ethanesulfonic acid (MES) and cultured by shaking overnight. The bacteria were collected at 4,000 RPM and centrifuged for 5 min. The bacteria suspension OD_600_ value was adjusted to 1.0 with 10 mM MgCl_2_ solution (containing 0.1 mM AS and 10 mM MES). The bacterial solution was injected into the lower epidermis of *N. benthamiana* leaves on three plants using a 1 mL syringe without a needle.

Leaves of *N. benthamiana* were collected 2 days after injection and incubated in GUS staining buffer (0.1 M pH 7.0 sodium phosphate, 10 mM pH 8.0 EDTA, 0.1% Triton X-100, 1 mM K_3_Fe (CN)_6_, 2 mM X-Gluc in dimethylformamide; Jefferson, 1987) for 24 h at 37°C. Then the staining buffer was removed and the leaves were washed with several changes of ethanol (up to 2 h per wash) until the chlorophyll fades. The discolored leaves were stored in 75% ethanol for photography.

### RNA Extraction and qRT-PCR Analysis

Leaves were collected from rice plants at the tillering stage or *N. benthamiana* 3 days after injection. RNA was extracted using KK Fast Plant Total RNA Kit (ZP405K, ZOMANBIO) according to the user manual. Total RNA of 2 μg was reversely transcribed into cDNA by using FastKing RT Kit (with gDNase; KR116, TIANGEN) according to the user manual. The cDNA product was diluted four times with ddH_2_O (RNase-free). A total of 20 μL volume, including 2 μL diluted cDNA was used in the qRT-PCR with Applied Biosystems 7500 Real-Time PCR System according to the user manual of Taq Pro Universal SYBR qPCR Master Mix (Q712, Vazyme). The *OsHYR* transcript was amplified using primers No. 13–14. The *OsSUT2* transcript was amplified using primers No. 15–16. The *OsNOG1* transcript was amplified using primers No. 17–18 (Supplementary Table 1). Each sample was amplified three times, and *OsUbq* (LOC4332169, primers No. 19–20, Supplementary Table 1) was used as a reference gene for rice. For *N. benthamiana*, the *GUS* gene was amplified using primers No. 21–22 (Supplementary Table 1). Each sample was amplified three times and *NbEF-1*α (Wang et al., 2018) (Nbv6.1trP76432, primers No. 23–24, Supplementary Table 1) was used as the reference gene. The results of qRT-PCR were analyzed by 2^–ΔΔ*Ct*^ method (Livak and Schmittgen, 2001).

### Treatment of Rice With Bacteria Strains

The PXO99^A^ and PH were spread on the NA medium (with ampicillin and spectinomycin added for PH-dt strains) and cultured for 2 days at 30°C. The bacteria were eluted with sterile water, shaken well, and the OD_600_ value of the suspension was adjusted to 1.0. For the leaf-cutting inoculation, sterile scissors were used to soak the bacteria solution and the tip of 2 cm of rice fully expanded leaves were cut, with three leaves per plant and no less than three plants each for phenotypic identification. The lesions were measured and photographed after 2 weeks (Zhang et al., 2013). For the injection inoculation, a common method to observe the infection degree of *Xanthomonas* or the expression changes of related genes of rice leaves (Tariq et al., 2018), a sterile syringe without a needle was used to inject bacterial fluid from the undersurface of the fully expanded leaves of no less than three rice plants at about 10 o’clock in the morning. For the spraying treatment, the bacteria solution was sprayed evenly on both sides of rice leaves in the 10 × 10 plant-plots with a hand-held sprayer every 3 days at about 10 o’clock in the morning from tillering stage to the filling stage of rice.

### Detection of Growth Curve of PH-dtNOG1 on Rice Leaves

The bacterial population density of PH-dtNOG1 was determined using the improved plate count method based on previous research (Bai et al., 2014). The whole leaves sprayed uniformly with the PH-dtNOG1 bacteria solution were collected. About 3 g leaves with 5 mL sterile water were ground into homogenate with sterilized mortar. A total of 50 μL homogenate was diluted 10, 100, and 1,000 times in sterile water, and then 50 μL was taken and coated on NA medium (ampicillin and spectinomycin added) solid plates and incubated at 30°C for 3 days. The plates with the number of colonies between 30 and 300 were selected for statistics. The final number of colony-forming unit per gram (CFU/g) was calculated according to this formula: CFU/g = A⋅F⋅100/W (A: counted colonies, F: dilution ratio, W: leaves weight). The final data was the mean of three experiments.

### Agronomic Traits Characterization

The agronomic traits of rice were investigated after the rice was fully mature. Plant height (the height of the ground part of the main tiller) and number of productive panicles (with more than 5 filled grains, NPP) were measured and recorded in the field. Panicle length, filled grain number per panicle (FGNPP), the number of empty grains per panicle, total grain number per panicle (TGNPP), setting rate, yield per plant, and 1,000-grain weight were measured after the fully mature panicles were harvested from the 6 × 6 plants area in the center of the 10 × 10 plants plot, with no less than 10 plants for each plot. The grain weight was measured after conventional drying of seeds. For JJ818, the agronomic traits were investigated independently in two growing seasons.

## Results

### Assembling of Designer Transcription Activator-Like Effectors and Response of *OsNOG1* to Designer Transcription Activator-Like Effector-NOG1

Rice growth or yield trait-related genes, *OsHYR* (Ambavaram et al., 2014), *OsSUT2* (Eom et al., 2011), and *OsNOG1* (Huo et al., 2017) were chosen as the target genes of dTALEs. The virulence-disarmed *Xoo* strain PH (Ji et al., 2016) was used as a device for delivering the dTALEs into rice plant cells. After analyzing the promoter sequences of the above-mentioned genes based on the reference genome of *japonica* rice variety Nip, along with considering factors including the number of repeat units of dTALEs to be assembled, the distance of target sites to the start codon and TSS of the target gene, target site sequence specificity in rice genome, and coverage with other transcription regulatory elements, three dTALEs targeting promoters of *OsHYR*, *OsSUT2*, and *OsNOG1* were assembled, respectively (Figure 2A). Each dTALE was transferred into the mutant *Xoo* strain PH, resulting in GEB strains PH-dtHYR, PH-dtSUT2, and PH-dtNOG1, respectively (Figures 2B,C).

Western blotting confirmed the stable expression of dTALEs in the bacteria PH, where the expected dTALE-NOG1, dTALE-HYR, and dTALE-SUT2 proteins with DYKDDDDK tag (133.2, 132.9, and 129.2 kDa, respectively) were clearly detected (Figure 2D and Supplementary Figure 1). By injecting Nip leaves with cell suspensions of the three GEB strains, we found that the expression of *OsNOG1* could be significantly induced by PH-dtNOG1, while the expression induction for the other two genes was not significant (Figures 2E–G). Therefore, *OsNOG1* and PH-dtNOG1 were used for subsequent experiments.

**FIGURE 3:**
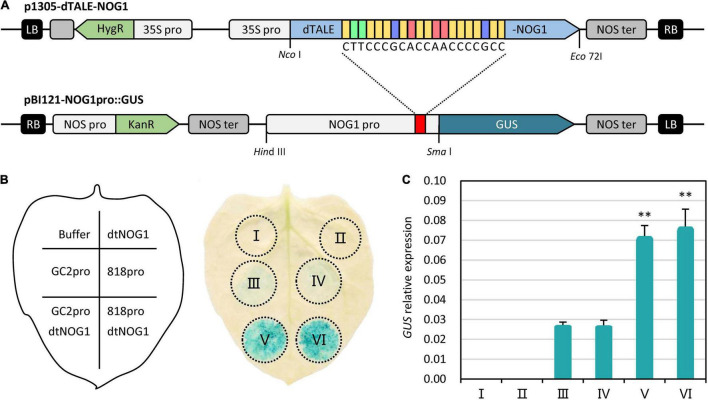
dTALE-NOG1 induced expression of GUS driven by *OsNOG1* promoter in *N. benthamiana*. **(A)** The activator vector, p1305-dTALE-NOG1, was generated by inserting the coding sequence of dTALE-NOG1 into *Nco*I and *Eco*72I sites of pCAMBIA1305. The reporter vector, pBI121-NOG1pro:GUS, was generated by inserting the promoter sequence of *OsNOG1* into *Hin*dIII and *Sma*I sites of pBI121. The dTALE target in *OsNOG1* promoter was shown by a red rectangle. **(B)** GUS expression assays on a *N. benthamiana* leaf with treatments of I–VI, corresponding to Buffer, p1305-dTALE-NOG1 (dtNOG1), pBI121-GC2NOG1pro:GUS (GC2pro, the promoter fragment was from GC2), pBI121-818NOG1pro:GUS (818pro, the promoter fragment was from JJ818), GC2pro with dtNOG1, 818pro with dtNOG1, respectively, as shown in the sketch leaf. **(C)** Relative expression of *GUS* in *N. benthamiana* detected by qRT-PCR. *NbEF-1*α (Nbv6.1trP76432) was used as the reference gene. Data values were represented as mean with error bars representing standard deviation (*n* = 3). The significance of the difference was tested by *t*-test. ^**^P < 0.01.

The function of dTALE’s transcriptional activation is often affected by various factors (Schreiber and Tissier, 2017). To further confirm the functional feasibility of dTALE-NOG1 before field application, the *GUS* gene was used as a reporter by cloning it into vectors downstream of the *OsNOG1* promotor from rice varieties GC2 or JJ818. The construct expressing dTALE-NOG1 was used as an activator (Figure 3A). The reporter and activator vectors were individually or co-transformed into *N. benthamiana* leaves by *Agrobacterium*-infiltration. GUS staining showed that the GUS expression, driven by *OsNOG1* promoter from either GC2 or JJ818 was very weak, but the expression was significantly enhanced in the presence of dTALE-NOG1 (Figure 3B). qRT-PCR showed that the relative expression of *GUS* was more than 2.5 times increased by dTALE-NOG1 in *N. benthamiana* (Figure 3C). Therefore, the dTALE-NOG1 was functional for *OsNOG1*, and the PH-dtNOG1 could be tested in the field for exploring its effects on rice.

### Rice Varieties Suitable for Testing the Effects of PH-dtNOG1 in Reprograming Gene Expression

To choose the appropriate rice varieties to test the effects of PH-dtNOG1 in reprograming gene expression and relevant agronomic trait change, we confirmed the conservativeness of the target gene *OsNOG1* in 4 varieties (Nip, GC2, JJ818, and R007) by sequencing. Nip was used as a sequence reference and control since it is the model variety of rice. GC2 is a high-yielding *indica* variety harboring the functional *OsNOG1* (Huo et al., 2017). JJ818 is a high-quality *japonica* rice variety suitable for planting in single-season rice-growing areas in North China, with good compact plant type, which was selected as the main rice material in this study for its convenience for spraying GEB and the potential to improve yield. R007 was used as a representative of *indica* rice for the yield test. The full length of *OsNOG1* in the above varieties was amplified and sequenced. Alignment analysis showed that the *OsNOG1* from JJ818 contains 12 SNPs and 2 Indels in the introns, 1 SNP in Exon 2, which does not change its coding amino acid (Threonine), and a 3-bp (GAG, one glutamate added for protein) insertion in exon 14 (Supplementary Figure 2), which was the same as that in the reported cDNA from rice introgression line SIL176, not affecting the function of NOG1 protein (Huo et al., 2017).

In the *OsNOG1* promoter from JJ818, there were 33 irregularly distributed SNPs and 6 Indels compared with that from GC2. The 12-bp deletion at −2,209 bp upstream of ATG for JJ818 was consistent with that in the introgression line SIL176 described previously, which plays a key role in regulating *OsNOG1* expression (Huo et al., 2017). The *Japonica* rice varieties JJ818 and Nip have 1 copy of the 12-bp Indel while the *indica* rice varieties GC2 and R007 have 2 copies of the 12-bp Indel. The 20 bp target site sequence (5′-CTTCCCGCACCAACCCCGCC-3′) of dTALE-NOG1 locates at -146 bp upstream of ATG was conserved in all the rice varieties tested (Figure 2B and Supplementary Figure 2). These results indicated that the *OsNOG1* gene was conserved and functional in the tested rice varieties, and dTALE-NOG1 could be used to target the promoter of *OsNOG1* in the rice varieties used in this study.

### PH-dtNOG1 Showed No Obvious Pathogenicity in Rice

**FIGURE 4:**
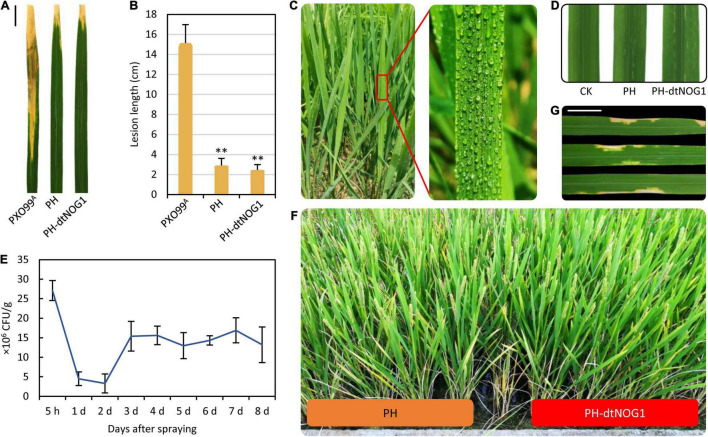
The response of rice variety JJ818 to *Xoo* strains PH and PH-dtNOG1. **(A,B)** PH and PH-dtNOG1 had no serious pathogenicity to JJ818 compared with the pathogenic strain PXO99^A^. The photos of leaves were taken 2 weeks post-inoculation. Scale, 2 cm. Data values were represented as the mean, with error bars representing standard deviation (*n* = 9). The significance of the difference was tested by *t*-test. ^**^P < 0.01. **(C)** Effect of spraying PH-dtNOG1 on rice leaves. **(D)** Injection of PH and PH-dtNOG1 cells into leaves of JJ818, without pathogenicity. Photos were taken 4 days after the injection. **(E)** The retention of PH-dtNOG1 in JJ818 leaves after once spraying treatment. Data values were represented as mean with error bars representing standard deviation (*n* = 3). **(F)** The plants of JJ818 in the filling stage after multiple-spraying treatment with PH or PH-dtNOG1. **(G)** After multiple-spraying PH-dtNOG1, some thin spots appeared at the late growth stage of rice plants. Scale, 1 cm.

The PH is a TALE-free mutant strain of PXO99^A^ that does not contain any TALE gene that plays key roles in rice disease (Ji et al., 2016) but still has a complete T3SS. This makes PH a good tool for dTALE delivery. Nevertheless, it is needed to determine the pathogenicity of the GEB PH-dtNOG1 before field application. Therefore, the wild-type *Xoo* strain PXO99^A^, the mutant strain PH, and the PH-dtNOG1 were inoculated on leaves of JJ818 and Nip by the cutting method (Zhang et al., 2013). After 2 weeks, the length of disease lesions was measured. Results showed that the disease lesion length caused by PXO99^A^ on JJ818 was 14.3 cm on average, while the lesions caused by PH and PH-dtNOG1 were only 2.9 and 2.5 cm in length, respectively, significantly shorter than that of PXO99^A^ (Figures 4A,B). Similar results were obtained on the Nip plants (Supplementary Figure 3). We further determined the pathogenicity of the *Xoo* strains by both injection and spraying methods, and results showed that no obvious disease phenotype was observed 4 days post-injection (Figure 4D) or spraying with PH or PH-dtNOG1 on JJ818 plants (Figure 4F). These observations confirmed that PH and PH-dtNOG1 had no obvious pathogenicity to JJ818 and Nip plants, and the mutant *Xoo* strain PH could be used to deliver dTALEs for regulating rice growth.

### Designer Transcription Activator-Like Effector-NOG1 Was Delivered Into Rice Leaves by Spraying PH-dtNOG1

Under natural conditions, infection, colonization, and reproduction of *Xanthomonas* on host plants mainly depend on the activation of host susceptibility (*S*) genes by TALEs secreted by *Xanthomonas*, while other genes targeted by TALEs tend to be off-target (Nowack et al., 2021). Since PH has no TALE genes, it is unable to promote the expression of *S* genes, and this would affect its viability in rice leaves. To clarify this issue, the retention density of PH-dtNOG1 on rice JJ818 leaves was measured after once uniformly spraying the bacteria (Figure 4C). Results showed that the number of bacteria was kept maximum at 5 h post-spraying and decreased rapidly in the following 1–2 days and reached the lowest on the second day. However, from the third day, the number of bacteria of PH-dtNOG1 began to increase and remained at a relatively stable level at about 15 × 10^6^ CFU/g, but still lower than the initial level (Figure 4E). These data showed that when the PH-dtNOG1 bacteria were transferred from the nutrient environment of the culture medium to the natural nutrient environment of leaves, a certain loss occurred, but the bacterial number would be recovered later. At the later stage of rice grain filling, some rice leaves with multiple-spraying treatment displayed tiny disease spots containing PH-dtNOG1, indicating that the GEB could be retained on rice leaves by spraying (Figure 4G).

**FIGURE 5:**
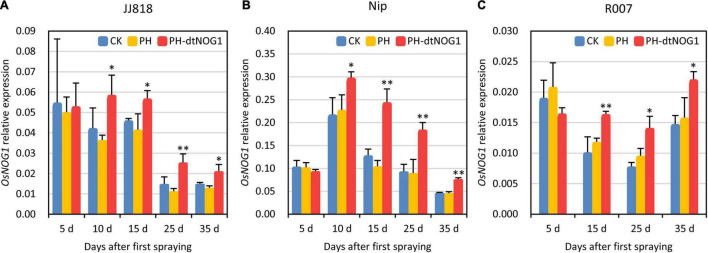
The expression of *OsNOG1* was induced by spraying PH-dtNOG1. Panels **(A–C)** showed the expression of *OsNOG1* in rice JJ818, Nip, and R007 untreated (CK, blue bars), treated with PH (yellow bars), and with PH-dtNOG1 (red bars), respectively, during the process of spraying treatments. *OsUbq* (LOC4332169) was used as the reference gene for qRT-PCR. Data values were represented as mean with error bars representing standard deviation (*n* = 3). The significance of the difference was tested by *t*-test. *0.01 < *P* < 0.05; ^**^P < 0.01.

During the tillering stage, the expression of *OsNOG1* was detected in rice leaves of JJ818, Nip, and R007 treated with spraying PH-dtNOG1 once every 3 days until the late filling stage in the field. On the fifth day after the first spraying (1 day before the third spraying), there was no significant change in the relative expression of *OsNOG1* in leaves sprayed with PH-dtNOG1 compared with the leaves sprayed with PH or without treatment (CK). The possible reason is that the first few days were colonization, recovery, and the reproduction stage of the GEB, when there was no enough bacteria to significantly affect the target gene *OsNOG1*. However, the relative expression of *OsNOG1* was significantly increased 10 days after the first spraying (2 days before fifth spraying) and continued to 35 days post the first spraying (Figures 5A–C) for JJ818, Nip, and R007. These results indicate that the GEB PH-dtNOG1 can colonize on rice leaves, deliver the dTALE-NOG1 into rice cells and induce the expression of *OsNOG1* when the plants were treated by simply repeatedly spraying the water solution of GEB without any additives.

### Rice Yield Was Increased by Spraying PH-dtNOG1

**FIGURE 6:**
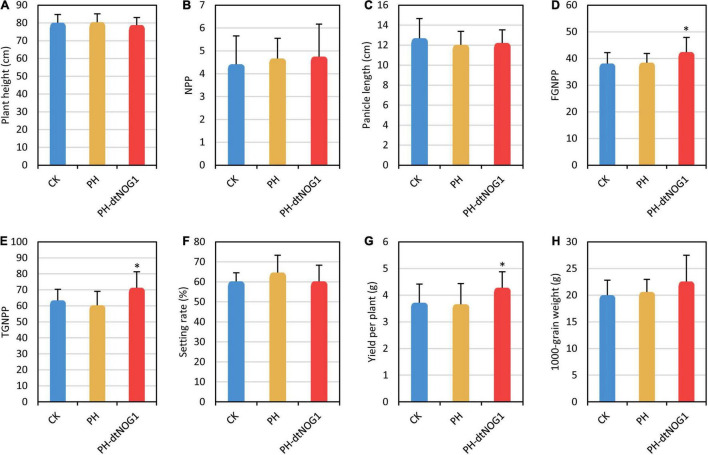
Rice yield was increased by spraying PH-dtNOG1. Panels **(A–H)** showed the plant height, number of productive panicles (NPP), panicle length, filled grain number per panicle (FGNPP), total grain number per panicle (TGNPP), setting rate, yield per plant and 1,000-grain weight of JJ818 untreated (CK, blue bars), and treated with PH (yellow bars) and PH-dtNOG1 (red bars), respectively. Data values were represented as mean with error bars representing standard deviation (*n* = 12). The significance of the difference was tested by *t*-test. *0.01 < *P* < 0.05.

Since panicle differentiation has been completed in the late tillering stage (Xu et al., 2020), the spraying treatment must be carried out before panicle initiation to increase the grain number by changing the expression of *OsNOG1*. Meanwhile, to ensure a stable number of PH-dtNOG1 cells on the leaves, the early tillering stage was selected as the time for first spraying and continued to the late filling stage. The spraying treatment was carried out once every 3 days. The agronomic characters of JJ818 were investigated when the rice grains were fully mature. Results showed that the FGNPP, TGNPP, yield per plant of JJ818 were significantly increased by treatment with PH-dtNOG1, compared with the control plants sprayed with PH and plants without treatment (CK), while the plant height, NPP, panicle length, setting rate, and 1,000-grain weight did not change significantly compared with the controls (Figures 6A–H). The FGNPP increased by 11.40%, the TGNPP increased by 12.51% and the yield per plant increased by 15.09% compared with CK (Supplementary Table 3). Taken together, the effect of PH-dtNOG1 on grain number promoted the increase of rice yield, although sporadic and small disease spots appeared on some leaves at late growth stage of the rice plants (Figure 4G).

Similar results have been obtained when other rice varieties were treated by spraying PH-dtNOG1. Compared with the plants without spraying, PH-dtNOG1 treated Nip showed no significant changes in plant height and NPP, but panicle length, FGNPP, TGNPP, and setting rate were significantly increased (Supplementary Figures 4A–H), among which FGNPP and TGNPP increased 17.21 and 12.10%, respectively. In addition, the yield per plant increased by 11.08%, and the 1,000-grain weight was also increased (Supplementary Table 3). Furthermore, PH-dtNOG1-treated R007, an *indica* variety, also showed a significant raise in TGNPP (Supplementary Figures 5C and Supplementary Table 3). These results demonstrated that regardless of the type of 1 or 2 copies of the 12-bp Indel in the *OsNOG1* promoter, the number of grains and yield could be increased by PH-dtNOG1 *via* induced expression of *OsNOG1*. Therefore, the use of the GEB PH-dtNOG1 targeting *OsNOG1* gene as the rice growth regulator can significantly promote rice yield traits without obvious pathogenicity or other side effects.

## Discussion

### The Genetically Engineered Bacteria-Designer Transcription Activator-Like Effector Strategy Is Feasible

**FIGURE 7:**
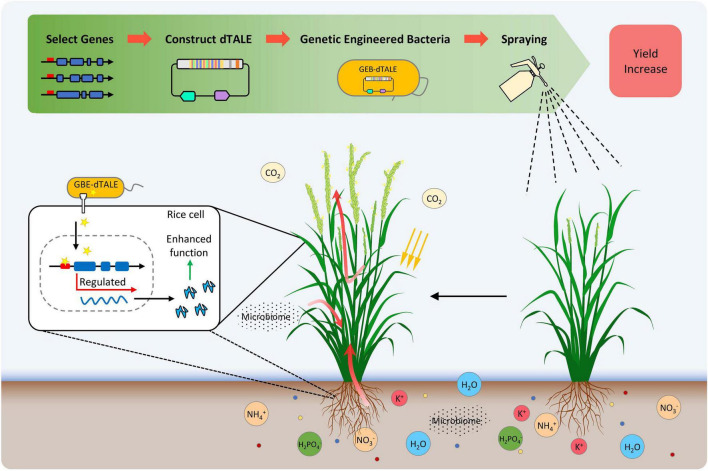
The GEB-dTALE strategy for yield increase by regulating crop plants growth. Four steps of GEB-dTALE strategy: (1) select target genes for dTALEs; (2) design and construct dTALE-expressing vectors; (3) transfer the dTALE construct into suitable delivery bacteria, resulting in genetically engineered bacteria (GEB); and (4) apply the GEB on crop plant through methods such as spraying. The dTALEs proteins (golden pentagram) are secreted by GEB and delivered into rice cells through T3SS of the GEB-dTALE cells. Afterward, dTALEs regulate the transcription of their target genes. Therefore, rice plants could obtain enhanced functions (red arrows) of root nutrient (circles of different colors) absorption, leaf photosynthesis, and carbon sequestration, source-sink transformation, effects with microbiome, etc., which may contribute greatly to rice yield increase. The function indicated by the dotted lines in the rice root needs further confirmation.

This proof-of-principle study proved that the mutant *Xoo* strain PH can be used as a device to deliver dTALEs, and the GEB strain PH-dtNOG1, which contains the artificially designed dTALE-NOG1 targeting the promoter of rice *OsNOG1* gene, had no obvious pathogenicity to rice, but could deliver the dTALE-NOG1 into rice cells and induce the expression of *OsNOG1*, and finally resulted in rice yield increase. Our experiments showed that the expression of *OsNOG1* was significantly up-regulated by spraying cell suspension of PH-dtNOG1 on rice plants during the tillering stage. For the *japonica* rice JJ818, the grain number per panicle of rice was increased by 12.51% and the yield per plant was increased by 15.09% compared with CK control. Similar results have been achieved on *japonica* rice Nip as well as the *indica* rice R007. Based on these findings, we proposed a new CAPME strategy for rice, namely GEB-dTALE strategy (Figure 7), which contains four steps: (1) choose appropriate genes as the targets of dTALEs; (2) design and construct the gene-specific dTALE’s expression vector, and perform functional verification of dTALEs; (3) transfer the dTALE-expressing construct into suitable delivery bacteria to generate GEB; and (4) apply the GEB on crop plant through methods such as spraying (Figure 7). The dTALEs should be secreted into the host cell through T3SS and should induce the target gene expression, resulting in changes in plant traits, such as improving nutrient absorption by plant root, leaf photosynthesis, source-sink transformation, effects of microbiome, and eventually achieve the increase of harvest (Figure 7).

### The Superiority and Inadequacy of Genetically Engineered Bacteria-Designer Transcription Activator-Like Effector Strategy

In recent decade, some new technologies for promoting plant growth have been developed and applied in agricultural production, such as microbial growth regulators (Wei and Jousset, 2017) and nano regulation technology (Gogos et al., 2012). A number of studies have shown that many bacteria from the genera *Bacillus*, *Pseudomonas*, *Enterobacter*, and *Streptomyces* can promote crop growth, yield increase, or resistance enhancement, and they have been developed as biofertilizers (Ngalimat et al., 2021). In principle, these biofertilizers regulate crop phenotypes through physiological metabolism, which is lacking in accuracy, and possibly cause physiological toxicity problems to plants (Chun et al., 2020). In contrast, the GEB-dTALE strategy could specifically regulate the target gene expression genetically and the associated agronomical traits by individual dTALE protein, which should be more accurate and do not cause physiological toxicity to plants. However, it is noteworthy that the GEB-dTALE strategy may face a pathogenic risk if a GEB strain is used for many years, and the usage of a genetically engineered microbe may lead to additional regulatory hurdles in some countries. Although the PH strain had no obvious pathogenic effect on cultivated rice varieties, small disease spots still appeared on some leaves at a later stage of spraying treatment (Figure 4G). It is needed to keep watch on the possibility of bacterial mutation in the field environment. Theoretically, bacteria do not cause disease, but with T3SS they can be used as dTALE-delivery tools. Therefore, other bacteria strains, such as endophytes, could be identified as better dTALE-delivery tools in future.

### The Genetically Engineered Bacteria-Designer Transcription Activator-Like Effector Method Has Great Potential for Optimization and Application

In the use of GEB-dTALE strategy, the selection of appropriate genes and target site sequences for dTALEs is particularly important. The *OsNOG1* gene used in this study is the only known single gene that plays a significant role in regulating rice yield traits, while other genes associated with rice growth or yield traits are involved in complex regulatory pathways regulating the corresponding phenotypes (Li et al., 2021). This might explain why the genes *OsHYR* or *OsSUT2* could not be induced by the constructed dTALEs (Figures 2E,F). However, studies have shown that a multiplex transcription activator-like effector activation (mTALE-Act) system can simultaneously activate the expression of four endogenous genes in plants by transgenic technology (Lowder et al., 2018). Therefore, it is theoretically possible to simultaneously up-regulate the expression of multiple genes through GEB-dTALE strategy. In addition, the dTALE can be modified by replacing the AD domain with transcription inhibitors (Bernstein et al., 2015) or just cascading a repression domain after dTALE (Mahfouz et al., 2012) or occupying key site of promoter (Sakono and Hayakawa, 2021) to inhibit or down-regulate gene expression. All of these provide a basis for using dTALE system to comprehensively regulate (induction or suppression or both) genes or gene networks, which makes the GEB-dTALE strategy have tremendous potential for further improvement. For a crop species, its gene resources are limited, and current crop breeding selection and cultivation approaches have almost pushed the crop yield to a plateau, which often accompanied by great time and material costs. In this context, the GEB-dTALE strategy provides a new idea and great potential for further exploration in the field of CAPME technology.

## Data Availability Statement

The original contributions presented in this study are included in the article/Supplementary Material, further inquiries can be directed to the corresponding authors.

## Author Contributions

KZ, CW, and ZJ conceived and designed the research. YT, CW, FW, ML, and YF performed the experiments. YT, KZ, and ZJ wrote the manuscript. All authors contributed to the article and approved the submitted version.

## Conflict of Interest

The authors declare that the research was conducted in the absence of any commercial or financial relationships that could be construed as a potential conflict of interest.

## Publisher’s Note

All claims expressed in this article are solely those of the authors and do not necessarily represent those of their affiliated organizations, or those of the publisher, the editors and the reviewers. Any product that may be evaluated in this article, or claim that may be made by its manufacturer, is not guaranteed or endorsed by the publisher.

## Footnotes

1
snapgene.com

2
https://www.dna.affrc.go.jp/PLACE/?action=newplace
