# Supplementary material for: Development of Designer Transcription Activator-Like Effector-Based Plant Growth Regulator for Higher Yield in Rice
Source: Front Plant Sci. 2022 Jun 14;13:924645. doi: 10.3389/fpls.2022.924645 (PMC9237611; doi:10.3389/fpls.2022.924645)
Supplement: Supplementary file 1 [file Data_Sheet_1.DOCX]

Supplementary Material

# Supplementary Figures


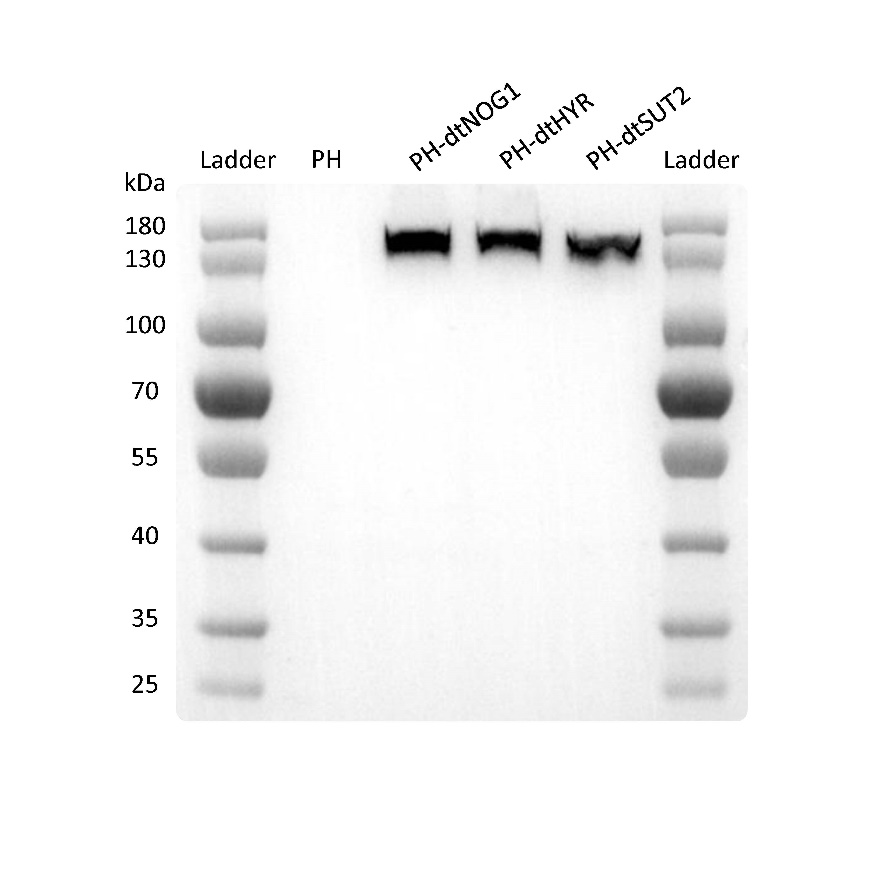


**Supplementary Figure 1.** Western blot of PH-dt strains.

Western blot of PH-dtNOG1, PH-dtHYR and PH-dtSUT2 using PH as the negative control. The dTALE-NOG1 protein expressed in PH-dtNOG1 was 133.2kDa. The dTALE-HYR protein expressed in PH-dtHYR was 132.9 kDa. The dTALE-SUT2 protein expressed in PH-dtSUT2 was 129.2 kDa. Ladder: PageRuler™ Prestained Protein Ladder (26616, Thermo Scientific).

**
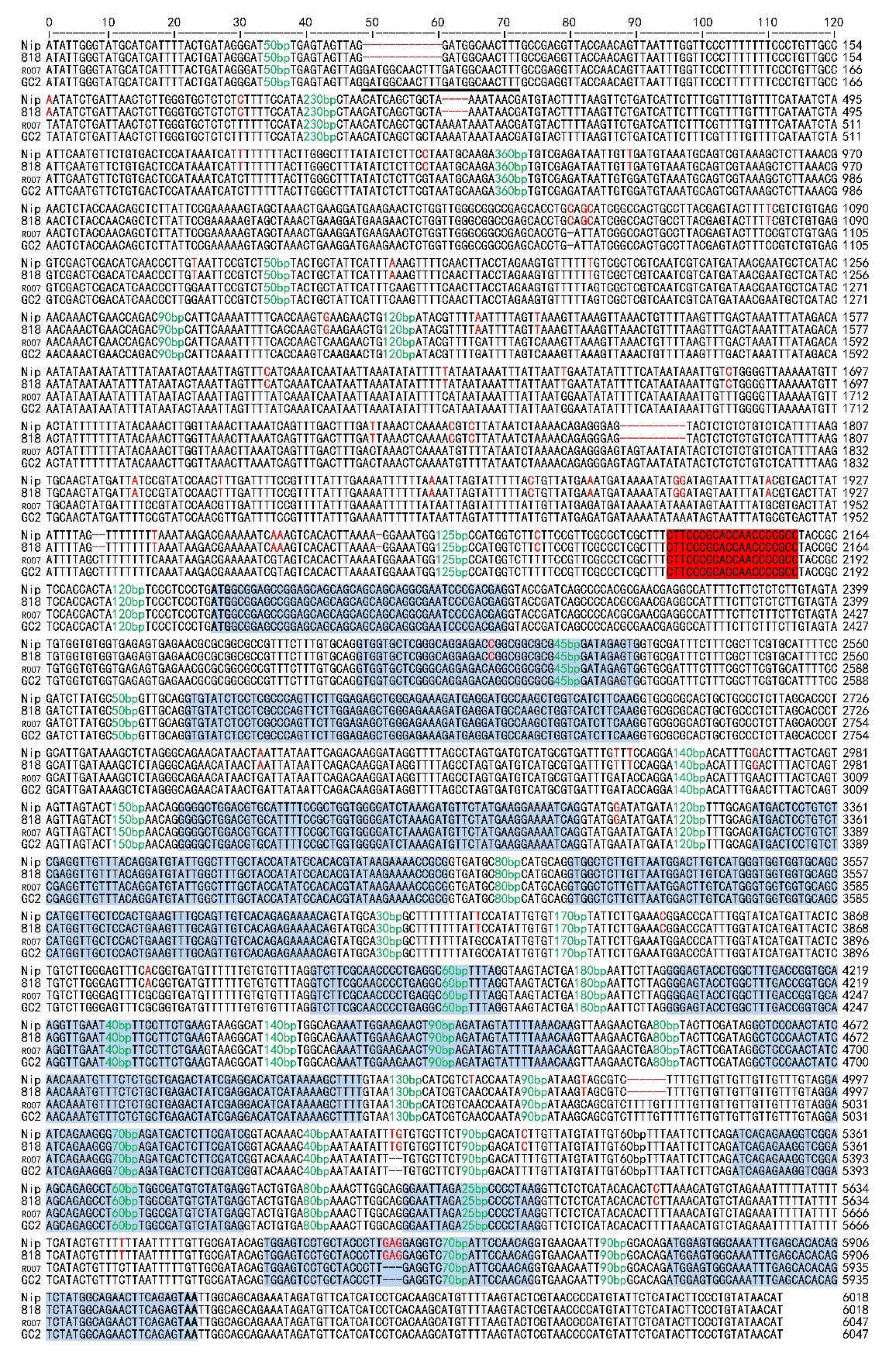
**

**Supplementary Figure 2.** Alignment of *OsNOG1* sequences from Nip, JJ818 (818), R007 and GC2.

Part of the undifferentiated sequences were replaced by green base-pair numbers. The difference sites were shown in red letters. The two closely connected copies of the 12-bp Indels in promoter were underlined. The dTALE target was shown red background at -146bp upstream of ATG. The start and end codons were shown in bold characters. The exons of *OsNOG1* were shown in light blue shading.


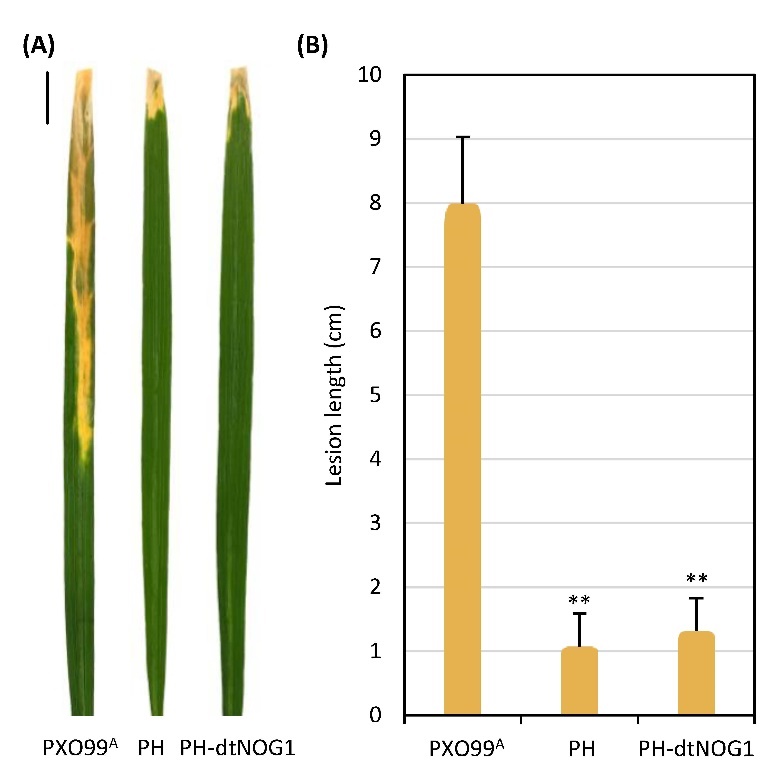


**Supplementary Figure 3.** PH and PH-dtNOG1 had no serious pathogenicity to rice Nip.

**(A-B)** PH and PH-dtNOG1 had no serious pathogenicity to rice Nip compared with the control PXO99^A^. Leaves photos were taken 2 weeks post inoculation. Scale = 1 cm. Data values were represented as mean with error bars representing standard deviation (n > 3). The significance of the difference was tested by t-test. **, P < 0.01.


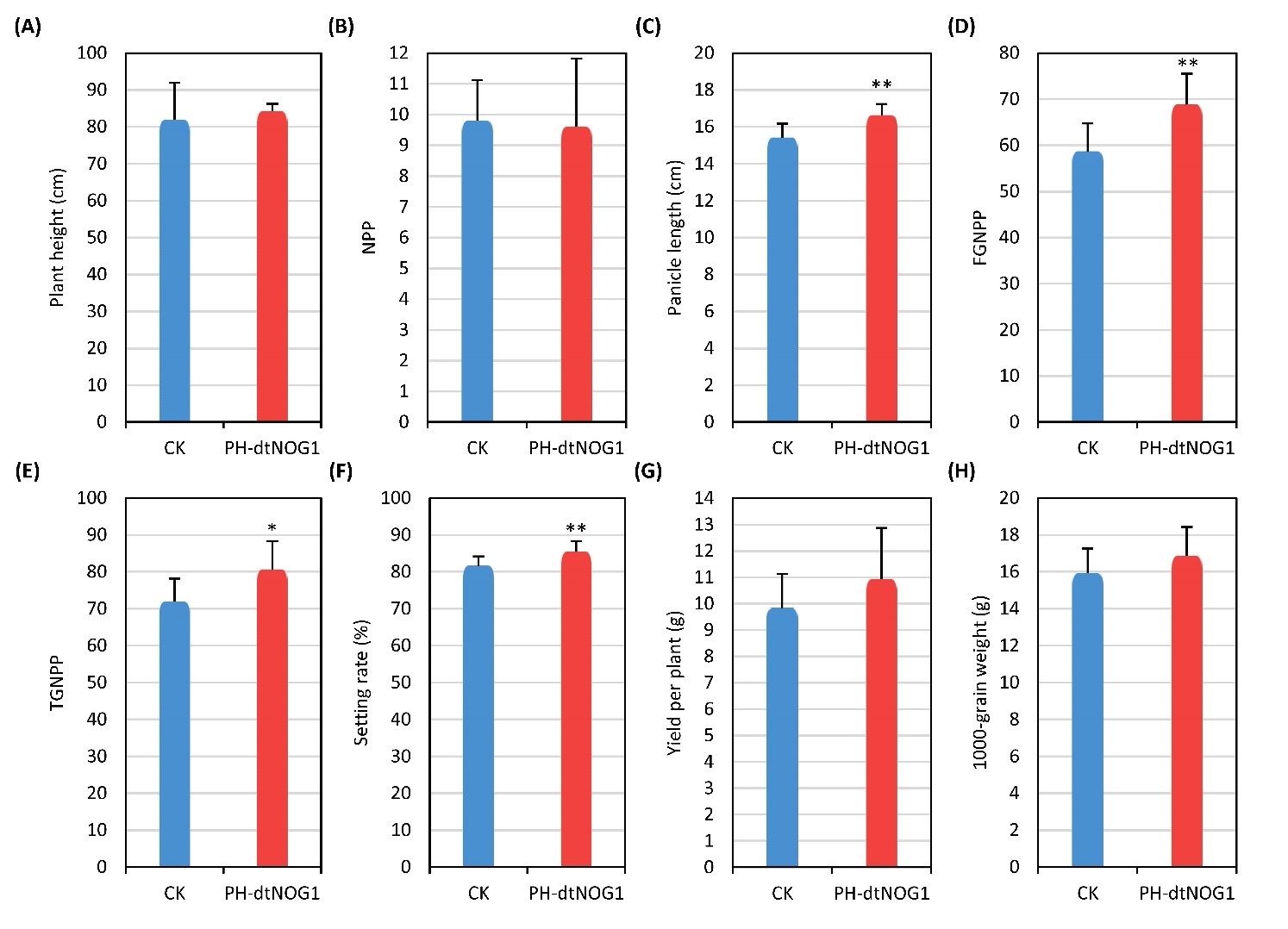


**Supplementary Figure 4.** Agronomic characters of Nip after spraying with PH-dtNOG1.

**(A-H)** showed the plant height, number of productive panicles (NPP), panicle length, filled grain number per panicle (FGNPP), total grain number per panicle (TGNPP), setting rate, yield per plant and 1000-grain weight of Nip treated with PH-dtNOG1(red bars) and untreated (blue bars). Data values were represented as mean with error bars representing standard deviation (n = 10). The significance of the difference was tested by t-test. *, 0.01 < P < 0.05. **, P < 0.01.


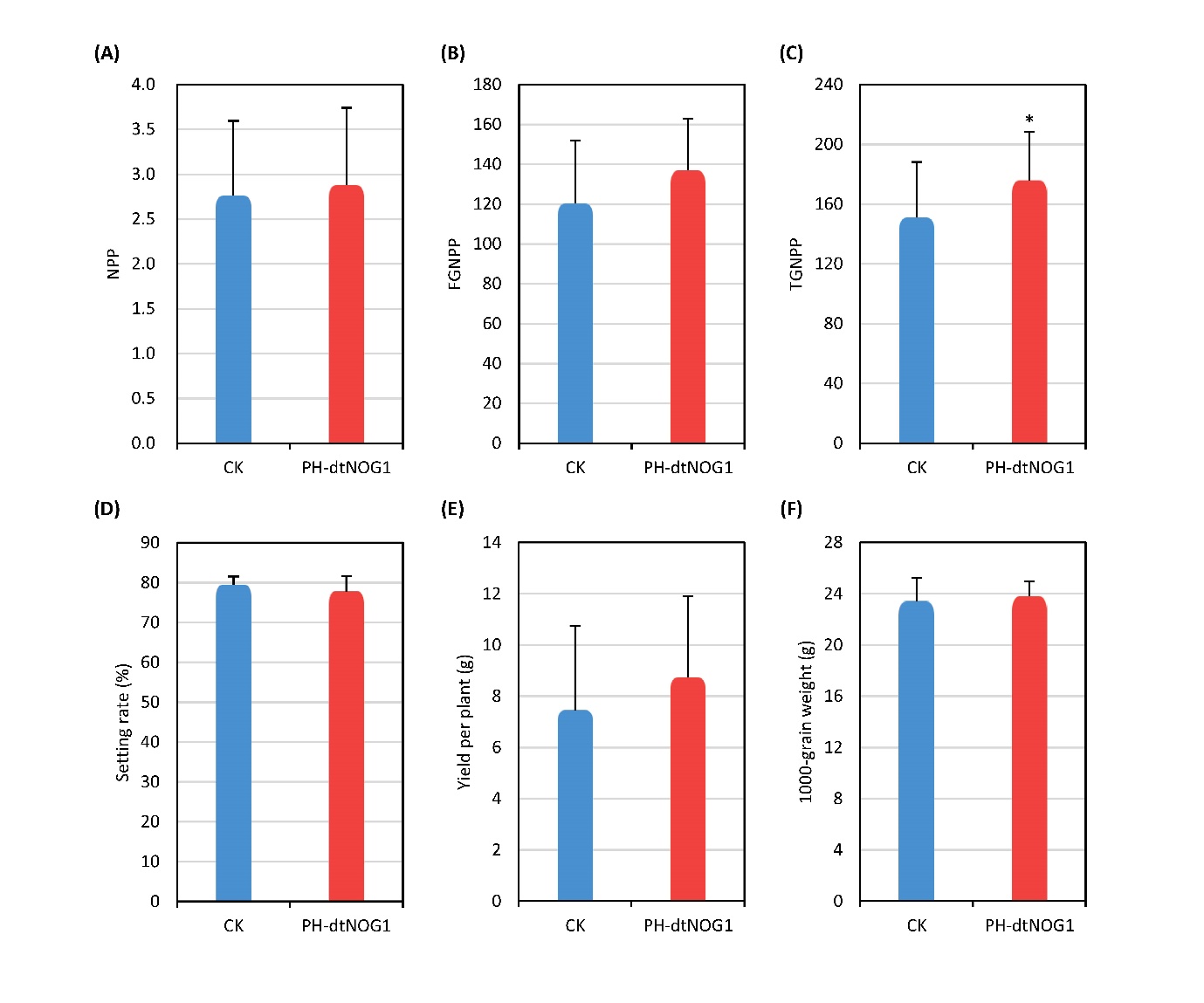


**Supplementary Figure 5.** Agronomic characters of R007 after spraying with PH-dtNOG1.

**(A-F)** showed the number of productive panicles (NPP), filled grain number per panicle (FGNPP), total grain number per panicle (TGNPP), setting rate, yield per plant and 1000-grain weight of R007 treated with PH-dtNOG1(red bars) and untreated (blue bars). Data values were represented as mean with error bars representing standard deviation (n = 17). The significance of the difference was tested by t-test. *, 0.01 < P < 0.05.

# Supplementary Tables

**Supplementary Table 1.** Primers used in this research.

| No. | Name | Sequence (5’-3’) | Used for |
| --- | --- | --- | --- |
| 1 | OsNOG1#-2352F | TATTTGTTTGGCACAACCGCA | Amplifying and sequencing of *OsNOG1* |
| 2 | OsNOG1#-1507F | AGCACTGCCCAAATGTTGGTT |  |
| 3 | OsNOG1#-269F | TGATCCCGAAGTGAAAACCGAT |  |
| 4 | OsNOG1#-1R | CAGGGAGGGACCCGAATTCC |  |
| 5 | OsNOG1#398F | CGCACTGCTGCCCTCTTAGC |  |
| 6 | OsNOG1#1148F | ACTGTTTTCTAACTTGGGGGCG |  |
| 7 | OsNOG1#1564F | CTGTCTTGGGAGTTTCACGGT |  |
| 8 | OsNOG1#1636R | AATCCAACACTAGCCTCAGGGG |  |
| 9 | OsNOG1#2283F | ACTTGCTGCGCATGCATTTATTA |  |
| 10 | OsNOG1#3765R | AGATGCTTTATTGCTTCGTGCGT |  |
| 11 | pBI121>OsNOG1#-2352F | GACCATGATTACGCCAAGCTTTATTTGTTTGGCACAACCGCA | Reporter vector construction |
| 12 | OsNOG1#-1R>pBI121 | ATAAGGGACTGACCACCCGGGCAGGGAGGGACCCGAATTC |  |
| 13 | HYR#487qF | CCGAGGGCTTGATGATGAG | qRT-PCR |
| 14 | HYR#615qR | CGTAAGCCCATTTCAGGAATG |  |
| 15 | SUT2#2192F | TGGTCTGATGCTGAACTCGG |  |
| 16 | SUT2#2562R | TTTCAACCCGACTAGCAGCC |  |
| 17 | NOG1#3128qF | TGGCGATGTCTATGAGGGAA |  |
| 18 | NOG1#3460qR | CGTCAGATGGGACTTGGAGC |  |
| 19 | Ubq-real-F | GCTCCGTGGCGGTATCAT |  |
| 20 | Ubq-real-R | CGGCAGTTGACAGCCCTAG |  |
| 21 | GUS#1286qF | CGGAAGCAACGCGTAAACTC |  |
| 22 | GUS#1404qR | ATAACGGTTCAGGCACAGCA |  |
| 23 | NbEF-1α-qF | GTATGCCTGGGTGCTTGAC |  |
| 24 | NbEF-1α-qR | ACAGGGACAGTTCCAATACCA |  |

**Supplementary Table 2.** Lesion length (cm) of JJ818 and Nip.

| JJ818 plants | PXO99^A^ | PH | PH-dtNOG1 |
| --- | --- | --- | --- |
| 1 | 17.3 | 1.8 | 2.4 |
| 2 | 12.0 | 2.9 | 2.9 |
| 3 | 13.1 | 3.8 | 2.0 |
| 4 | 14.4 | 2.6 | 2.0 |
| 5 | 15.4 | 3.8 | 2.9 |
| 6 | 15.7 | 2.3 | 2.0 |
| 7 | 17.6 | 3.2 | 2.3 |
| 8 | 14.6 | 3.4 | 3.5 |
| 9 | 16.2 | 2.4 | 2.1 |
| Average | 15.1 | 2.9 | 2.5 |
| Standard deviation | 1.8 | 0.7 | 0.5 |

| Nip plants | PXO99^A^ | PH | PH-dtNOG1 |
| --- | --- | --- | --- |
| 1 | 7.5 | 1.7 | 0.8 |
| 2 | 6.8 | 0.7 | 1.6 |
| 3 | 7.4 | 1.3 | 1.5 |
| 4 | 6.3 | 2.0 | 0.7 |
| 5 | 8.4 | 0.8 | 2.1 |
| 6 | 8.8 | 0.8 | 0.9 |
| 7 | 8.6 | 0.6 | 1.7 |
| 8 | 9.5 | 0.5 | 1.7 |
| 9 | 8.6 | 1.2 | 0.8 |
| Average | 8.0 | 1.1 | 1.3 |
| Standard deviation | 1.8 | 0.7 | 0.5 |

Lesion length was measured after two weeks of inoculation.

**Supplementary Table 3** Statistical analysis of agronomic traits of rice treated with spraying PH-dtNOG1.

| Variety | Subspecies | Treatment | Plant height  (cm) | NPP | Panicle length  (cm) | FGNPP | TGNPP | Setting rate  (%) | Yield per plant  (g) | 1000-grain weight (g) |
| --- | --- | --- | --- | --- | --- | --- | --- | --- | --- | --- |
| JJ818 | *Japonica* | CK | 80.13±4.68 | 4.42±1.24 | 12.70±1.97 | 38.08±4.14 | 63.33±7.01 | 60.27±4.24 | 3.71±0.71 | 19.99±2.81 |
|  |  | PH | 80.42±4.77 | 4.67±0.89 | 12.04±1.34 | 38.42±3.48 | 60.33±8.75 | 64.59±8.71 | 3.66±0.78 | 20.60±2.35 |
|  |  | dtNOG1 | 78.83±4.22 | 4.75±1.42 | 12.22±1.31 | 42.42±5.50* | 71.25±10.01* | 60.19±8.15 | 4.27±0.61* | 22.56±4.94 |
| Nip | *Japonica* | CK | 81.96±10.04 | 9.80±1.32 | 15.41±0.76 | 58.70±6.06 | 71.90±6.24 | 81.57±2.55 | 9.84±1.28 | 15.92±1.33 |
|  |  | dtNOG1 | 84.19±2.10 | 9.60±2.22 | 16.63±0.61** | 68.80±6.71** | 80.60±7.66* | 85.39±2.82** | 10.93±1.94 | 16.85±1.57 |
| R007 | *Indica* | CK | — | 2.76±0.83 | — | 120.47±31.33 | 151.06±37.10 | 79.51±2.09 | 7.46±3.28 | 23.43±1.82 |
|  |  | dtNOG1 | — | 2.88±0.86 | — | 136.88±26.06 | 175.94±32.47* | 77.83±3.78 | 8.74±3.18 | 23.83±1.14 |
| Increase scope | | — | — | — | — | 11.40-17.21% | 12.10-16.47% | — | 11.08-17.16% | — |

Results showed the agronomic traits data of three rice varieties treated with spraying PH-dtNOG1 (dtNOG1), including plant height, number of productive panicles (NPP), panicle length, filled grain number per panicle (FGNPP), total grain number per panicle (TGNPP), setting rate, yield per plant and 1000-grain weight. Data values were represented as mean ± standard deviation (n ≥ 10). The significance of the difference was tested by t-test. *, 0.01 < P < 0.05. **, P < 0.01. Increase scope were calculated compared with CK.

**Supplementary Table 4** Agronomic characters data of JJ818 after spraying with PH-dtNOG1.

| CK | Plant height  (cm) | NPP | Panicle length (cm) | | | | | | | FGNPP | TGNPP | Setting rate  (%) | Yield per plant  (g) | 1000-grain weight  (g) |
| --- | --- | --- | --- | --- | --- | --- | --- | --- | --- | --- | --- | --- | --- | --- |
| 1 | 79.0 | 5 | 12.6 | 11.1 | 13.4 | 15.3 | 14.6 |  |  | 36 | 57 | 63.16 | 3.58 | 19.75 |
| 2 | 79.0 | 3 | 13.8 | 14.5 | 16.3 |  |  |  |  | 40 | 72 | 55.56 | 3.3 | 23.72 |
| 3 | 77.5 | 4 | 13.6 | 14.7 | 15.5 | 10.4 |  |  |  | 37 | 56 | 66.07 | 3.49 | 15.87 |
| 4 | 76.0 | 5 | 11.6 | 12.0 | 9.5 | 10.5 | 12.7 |  |  | 35 | 63 | 55.56 | 3.6 | 23.08 |
| 5 | 82.0 | 4 | 12.8 | 13.3 | 13.5 | 12.0 |  |  |  | 29 | 52 | 55.77 | 4.4 | 16.55 |
| 6 | 73.5 | 6 | 11.2 | 13.4 | 9.4 | 11.0 | 12.2 | 11.4 |  | 40 | 72 | 55.56 | 4.83 | 17.50 |
| 7 | 85.0 | 4 | 14.3 | 13.8 | 15.2 | 13.3 |  |  |  | 44 | 70 | 62.86 | 3.87 | 22.50 |
| 8 | 81.0 | 7 | 13.0 | 14.0 | 14.8 | 12.2 | 9.8 | 10.4 | 9.7 | 42 | 64 | 65.63 | 4.98 | 23.14 |
| 9 | 80.0 | 4 | 9.9 | 11.7 | 10.5 | 12.8 |  |  |  | 41 | 66 | 62.12 | 3.72 | 18.04 |
| 10 | 88.5 | 5 | 15.4 | 16.9 | 13.2 | 10.0 | 9.8 |  |  | 38 | 68 | 55.88 | 3.02 | 19.47 |
| 11 | 86.0 | 3 | 15.5 | 14.1 | 13.5 |  |  |  |  | 41 | 66 | 62.12 | 2.91 | 18.13 |
| 12 | 74.0 | 3 | 10.0 | 12.2 | 14.6 |  |  |  |  | 34 | 54 | 62.96 | 2.82 | 22.08 |
| Average | 80.1 | 4.4 | 12.7 | | | | | | | 38.1 | 63.3 | 60.27 | 3.71 | 19.99 |
| SD | 4.7 | 1.2 | 2.0 | | | | | | | 4.1 | 7.0 | 4.24 | 0.71 | 2.81 |
|  |  |  |  |  |  |  |  |  |  |  |  |  |  |  |
| PH | Plant height  (cm) | NPP | Panicle length (cm) | | | | | | | FGNPP | TGNPP | Setting rate  (%) | Yield per plant  (g) | 1000-grain weight  (g) |
| 1 | 79.5 | 5 | 10.9 | 13.9 | 11.8 | 11.7 | 10.1 |  |  | 38 | 56 | 67.86 | 4.38 | 23.30 |
| 2 | 78.0 | 5 | 13.0 | 12.3 | 11.5 | 10.0 | 12.0 |  |  | 37 | 52 | 71.15 | 3.65 | 19.73 |
| 3 | 74.0 | 4 | 13.2 | 10.5 | 14.1 | 12.2 |  |  |  | 37 | 74 | 50.00 | 3.24 | 22.19 |
| 4 | 74.0 | 6 | 9.4 | 13.2 | 11.8 | 13.7 | 12.4 | 13.6 |  | 42 | 62 | 67.74 | 3.97 | 15.82 |
| 5 | 75.0 | 5 | 11.8 | 12.7 | 13.0 | 14.1 | 12.2 |  |  | 41 | 60 | 68.33 | 4.56 | 22.35 |
| 6 | 82.0 | 4 | 11.6 | 11.7 | 11.6 | 13.6 |  |  |  | 42 | 58 | 72.41 | 3.71 | 22.22 |
| 7 | 81.0 | 5 | 9.4 | 12.7 | 14.2 | 10.6 | 10.9 |  |  | 40 | 59 | 67.80 | 4.16 | 20.59 |
| 8 | 88.0 | 4 | 12.1 | 8.8 | 11.4 | 10.4 |  |  |  | 39 | 53 | 73.58 | 2.71 | 17.60 |
| 9 | 79.5 | 3 | 11.5 | 11.5 | 10.2 |  |  |  |  | 40 | 61 | 65.57 | 2.24 | 18.67 |
| 10 | 87.0 | 4 | 11.7 | 10.3 | 13.3 | 13.4 |  |  |  | 31 | 62 | 50.00 | 2.75 | 22.18 |
| 11 | 81.5 | 6 | 14.1 | 12.9 | 12.4 | 13.3 | 10.4 | 11.4 |  | 33 | 48 | 68.75 | 3.87 | 19.64 |
| 12 | 85.5 | 5 | 13.0 | 13.4 | 13.1 | 11.4 | 12.8 |  |  | 41 | 79 | 51.90 | 4.66 | 22.96 |
| Average | 80.4 | 4.7 | 12.0 | | | | | | | 38.4 | 60.3 | 64.59 | 3.66 | 20.60 |
| SD | 4.8 | 0.9 | 1.3 | | | | | | | 3.5 | 8.8 | 8.71 | 0.78 | 2.35 |
|  |  |  |  |  |  |  |  |  |  |  |  |  |  |  |
| PH-dtNOG1 | Plant height  (cm) | NPP | Panicle length (cm) | | | | | | | FGNPP | TGNPP | Setting rate  (%) | Yield per plant  (g) | 1000-grain weight  (g) |
| 1 | 73.0 | 7 | 12.8 | 10.3 | 12.2 | 10.9 | 10.8 | 12.7 | 10.7 | 38 | 62 | 61.29 | 4.42 | 16.43 |
| 2 | 73.0 | 5 | 11.6 | 12.9 | 13.8 | 15.2 | 11.9 |  |  | 35 | 74 | 47.30 | 4.13 | 23.47 |
| 3 | 79.5 | 4 | 11.5 | 11.5 | 9.7 | 9.6 |  |  |  | 36 | 47 | 76.60 | 3.98 | 27.83 |
| 4 | 73.0 | 4 | 11.4 | 12.9 | 11.3 | 11.8 |  |  |  | 53 | 81 | 65.43 | 4.37 | 19.86 |
| 5 | 81.0 | 3 | 13.0 | 12.1 | 11.3 |  |  |  |  | 42 | 69 | 60.87 | 3.91 | 30.79 |
| 6 | 79.0 | 6 | 13.5 | 10.2 | 12.6 | 13.6 | 10.3 | 13.1 |  | 47 | 69 | 68.12 | 4.73 | 16.65 |
| 7 | 82.5 | 4 | 13.1 | 9.6 | 13.5 | 13.4 |  |  |  | 36 | 69 | 52.17 | 3.24 | 22.50 |
| 8 | 84.0 | 4 | 14.0 | 14.9 | 12.5 | 12.0 |  |  |  | 46 | 81 | 56.79 | 4.55 | 24.86 |
| 9 | 82.0 | 6 | 12.2 | 10.0 | 14.7 | 12.1 | 12.6 | 12.5 |  | 45 | 67 | 67.16 | 5.25 | 19.44 |
| 10 | 76.5 | 3 | 11.9 | 13.7 | 13.1 |  |  |  |  | 47 | 81 | 58.02 | 4.17 | 29.79 |
| 11 | 78.0 | 4 | 13.5 | 12.6 | 13.0 | 13.4 |  |  |  | 43 | 81 | 53.09 | 3.41 | 21.45 |
| 12 | 84.5 | 7 | 11.8 | 13.4 | 12.0 | 11.2 | 11.7 | 11.3 | 11.5 | 41 | 74 | 55.41 | 5.12 | 17.66 |
| Average | 78.8 | 4.8 | 12.2 | | | | | | | 42.4 | 71.3 | 60.19 | 4.27 | 22.56 |
| SD | 4.2 | 1.4 | 1.3 | | | | | | | 5.5 | 10.0 | 8.15 | 0.61 | 4.94 |

Abbreviations: NPP: Number of Productive Panicles; FGNPP: Filled Grain Number Per Panicle; TGNPP: Total Grain Number Per Panicle; SD: Standard Deviation.

**Supplementary Table 5** Agronomic characters data of Nip after spraying with PH-dtNOG1.

| CK | Plant height  (cm) | NPP | Panicle length  (cm) | FGNPP | TGNPP | Setting rate  (%) | Yield per plant  (g) | 1000-grain weight  (g) |
| --- | --- | --- | --- | --- | --- | --- | --- | --- |
| 1 | 68.1 | 10 | 14.8 | 56 | 66 | 84.85 | 9.32 | 14.82 |
| 2 | 68.5 | 10 | 14.2 | 52 | 68 | 76.47 | 8.04 | 14.08 |
| 3 | 92.0 | 11 | 14.7 | 56 | 68 | 82.35 | 10.95 | 16.15 |
| 4 | 86.2 | 9 | 16.1 | 70 | 83 | 84.34 | 9.34 | 17.20 |
| 5 | 92.1 | 9 | 15.2 | 57 | 70 | 81.43 | 11.69 | 13.92 |
| 6 | 68.0 | 11 | 16.6 | 53 | 67 | 79.10 | 10.56 | 17.52 |
| 7 | 91.7 | 7 | 16.3 | 68 | 83 | 81.93 | 8.73 | 17.39 |
| 8 | 84.4 | 9 | 15.2 | 56 | 70 | 80.00 | 8.23 | 15.56 |
| 9 | 82.5 | 11 | 15.4 | 57 | 70 | 81.43 | 10.37 | 15.69 |
| 10 | 86.1 | 11 | 15.6 | 62 | 74 | 83.78 | 11.16 | 16.83 |
| Average | 82.0 | 9.8 | 15.4 | 58.7 | 71.9 | 81.57 | 9.84 | 15.92 |
| SD | 10.0 | 1.3 | 0.8 | 6.1 | 6.2 | 2.55 | 1.28 | 1.33 |
|  |  |  |  |  |  |  |  |  |
| PH-dtNOG1 | Plant height  (cm) | NPP | Panicle length  (cm) | FGNPP | TGNPP | Setting rate  (%) | Yield per plant  (g) | 1000-grain weight  (g) |
| 1 | 83.1 | 5 | 15.5 | 79 | 86 | 91.86 | 9.19 | 19.22 |
| 2 | 85.2 | 9 | 17.3 | 64 | 75 | 85.33 | 10.82 | 17.00 |
| 3 | 86.0 | 9 | 17.1 | 81 | 96 | 84.38 | 12.00 | 16.75 |
| 4 | 85.0 | 9 | 16.0 | 67 | 78 | 85.90 | 8.72 | 15.77 |
| 5 | 81.4 | 10 | 17.2 | 65 | 78 | 83.33 | 9.72 | 15.94 |
| 6 | 84.4 | 11 | 16.1 | 71 | 85 | 83.53 | 14.18 | 19.69 |
| 7 | 84.5 | 14 | 16.5 | 61 | 72 | 84.72 | 13.76 | 16.40 |
| 8 | 80.1 | 10 | 17.0 | 62 | 70 | 88.57 | 8.77 | 14.62 |
| 9 | 87.0 | 10 | 17.1 | 69 | 83 | 83.13 | 11.38 | 17.24 |
| 10 | 85.2 | 9 | 16.5 | 69 | 83 | 83.13 | 10.73 | 15.83 |
| Average | 84.2 | 9.6 | 16.6 | 68.8 | 80.6 | 85.39 | 10.93 | 16.85 |
| SD | 2.1 | 2.2 | 0.6 | 6.7 | 7.7 | 2.82 | 1.94 | 1.57 |

Abbreviations: NPP: Number of Productive Panicles; FGNPP: Filled Grain Number Per Panicle; TGNPP: Total Grain Number Per Panicle; SD: Standard Deviation.

**Supplementary Table 6** Agronomic characters data of R007 after spraying with PH-dtNOG1.

| CK | NPP | FGNPP | TGNPP | Setting rate (%) | Yield per plant (g) | 1000-grain weight (g) |
| --- | --- | --- | --- | --- | --- | --- |
| 1 | 3 | 146 | 188 | 77.66 | 12.02 | 25.41 |
| 2 | 2 | 73 | 93 | 78.49 | 3.01 | 22.13 |
| 3 | 2 | 96 | 122 | 78.69 | 4.26 | 22.07 |
| 4 | 3 | 121 | 150 | 80.67 | 8.69 | 24.48 |
| 5 | 4 | 104 | 138 | 75.36 | 10.86 | 24.19 |
| 6 | 3 | 164 | 200 | 82.00 | 8.66 | 23.92 |
| 7 | 3 | 96 | 124 | 77.42 | 5.30 | 26.37 |
| 8 | 3 | 64 | 80 | 80.00 | 4.30 | 18.78 |
| 9 | 2 | 141 | 172 | 81.98 | 7.27 | 25.69 |
| 10 | 2 | 78 | 100 | 78.00 | 3.65 | 23.40 |
| 11 | 2 | 138 | 175 | 78.86 | 5.06 | 24.10 |
| 12 | 3 | 153 | 185 | 82.70 | 11.19 | 24.81 |
| 13 | 2 | 137 | 171 | 80.12 | 4.00 | 21.51 |
| 14 | 3 | 149 | 182 | 81.87 | 11.01 | 23.48 |
| 15 | 5 | 108 | 138 | 78.26 | 12.70 | 22.09 |
| 16 | 2 | 122 | 157 | 77.71 | 5.64 | 23.02 |
| 17 | 3 | 158 | 193 | 81.87 | 9.16 | 22.79 |
| Average | 2.8 | 120.5 | 151.1 | 79.51 | 7.46 | 23.43 |
| SD | 0.8 | 31.3 | 37.1 | 2.09 | 3.28 | 1.82 |
|  |  |  |  |  |  |  |
| PH-dtNOG1 | NPP | FGNPP | TGNPP | Setting rate (%) | Yield per plant (g) | 1000-grain weight (g) |
| 1 | 4 | 139 | 173 | 80.35 | 6.31 | 23.20 |
| 2 | 4 | 153 | 191 | 80.10 | 11.81 | 26.01 |
| 3 | 2 | 96 | 118 | 81.36 | 4.28 | 22.29 |
| 4 | 3 | 102 | 127 | 80.31 | 7.06 | 23.00 |
| 5 | 2 | 137 | 177 | 77.40 | 9.92 | 24.99 |
| 6 | 3 | 152 | 189 | 80.42 | 10.98 | 23.97 |
| 7 | 3 | 147 | 189 | 77.78 | 7.99 | 23.43 |
| 8 | 2 | 132 | 187 | 70.59 | 9.12 | 23.69 |
| 9 | 3 | 121 | 156 | 77.56 | 7.83 | 23.94 |
| 10 | 3 | 112 | 155 | 72.26 | 6.50 | 24.25 |
| 11 | 2 | 163 | 201 | 81.09 | 8.00 | 24.46 |
| 12 | 3 | 208 | 269 | 77.32 | 13.64 | 24.98 |
| 13 | 5 | 139 | 173 | 80.35 | 16.70 | 23.96 |
| 14 | 3 | 131 | 175 | 74.86 | 9.61 | 24.45 |
| 15 | 2 | 120 | 168 | 71.43 | 5.19 | 23.17 |
| 16 | 2 | 152 | 181 | 83.98 | 5.79 | 24.33 |
| 17 | 3 | 123 | 162 | 75.93 | 7.80 | 21.02 |
| Average | 2.9 | 136.9 | 175.9 | 77.83 | 8.74 | 23.83 |
| SD | 0.9 | 26.1 | 32.5 | 3.78 | 3.18 | 1.14 |

Abbreviations: NPP: Number of Productive Panicles; FGNPP: Filled Grain Number Per Panicle; TGNPP: Total Grain Number Per Panicle; SD: Standard Deviation.
